# Partial duplication of vas deferens: How important is it?

**DOI:** 10.4103/0971-9261.72437

**Published:** 2010

**Authors:** Ayşe Karaman, İbrahim Karaman, Beytullah Yağız, Yusuf Hakan Çavuşoğlu

**Affiliations:** Department of Pediatric Surgery, Dr. Sami Ulus Children’s Hospital, Ankara, Turkey

**Keywords:** Duplication, undescended testis, vas

## Abstract

This study reports a 1-year-old boy with unilateral partial duplication of vas deferens, diagnosed during surgery for undescended testis. Pediatric surgeons need to be aware of this kind of anomaly in order to avoid injury to this vital structure.

## INTRODUCTION

Partial duplication of the vas deferens at the inguinal canal level is an extremely rare congenital anomaly.[1] However, this anomaly should always be kept in the mind of a surgeon performing inguinal procedures. We are reporting here a case of partial duplication of the vas deferens discovered during an undescended testis surgery.

## CASE REPORT

**Figure 1:**
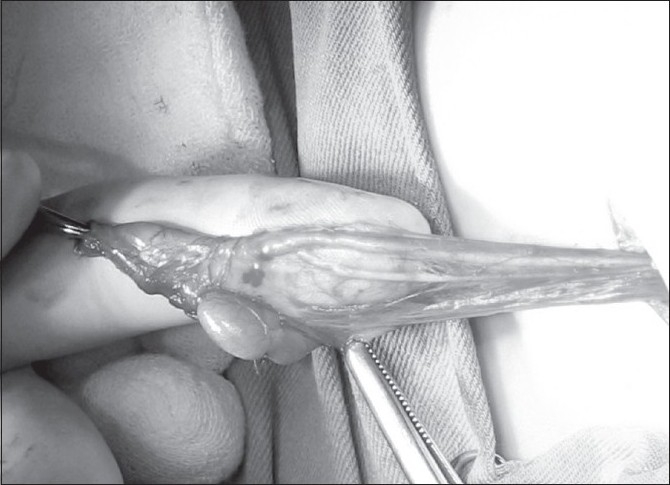
Intraoperative photograph showing two separate, different sized vas deferens, which unite to form a single structure 1 cm above the epididymis

A 1-year-old boy presented with bilateral undescended testes. On physical examination, both testicles were not found in the scrotum or inguinal canal. The ultrasound examination revealed a structure of 11 × 6 mm in size with smooth contours located proximally in the left inguinal canal and appeared compatible with left testis, while the right testis could not be identified in the abdomen or inguinal canal. On inguinal exploration, both testicles were found in the proximal level of the each inguinal canal. The epididymal attachments of the right testis appeared normal at the cranial portion, but the body and tail of the epididymis were found to be free and unattached. An orchiopexy was performed. During the dissection of the hernia sac from adjacent cord structures on the left, we isolated two separate, different sized vas deferens which united to form a single structure 1 cm above the epididymis [Figure 1]. The left testis and the epididymis appeared normal, and an orchiopexy was performed. The patient’s postoperative course was uneventful.

The postoperative ultrasonography did not identify any abnormality involving the collecting system or the structure of the kidney. The sweat chloride test for cystic fibrosis was found to be normal. At 1-year follow-up, scrotum and testicles were judged as normal bilaterally.

## DISCUSSION

The overall incidence of vas deferens anomalies in the general population is estimated to be less than 0.05%, and can be categorized as the absent, ectopic, hypoplastic, and duplicated.[1] Duplication of the vas deferens is a rare anomaly with only a few cases reported in the literature.[1–9]

The embryologic etiology of this anomaly is incompletely understood. It has been suggested that the duplication of the vas deferens may be due to duplication of the fetal mesonephric system. According to this theory, vas deferens develops from the central portion of the mesonephric duct which is termed as “proximal vas precursor.” Duplication of the proximal vas precursor presumably gives rise to the duplication of vas deferens.[10] According to another theory, transversal division of Wolffian duct during organogenesis causes the duplication of vas deferens.[11]

Operative injury to the vas deferens may result in obstruction of the vas, with diversion of spermatozoa to the testicular lymphatics. This breach of the blood–testis barrier produces an antigenic challenge, with the formation of spermatic autoagglutinating antibodies that ultimately leads to infertility.[12] Identification of the vas deferens during exploration of the spermatic cord for a hernia sac prevents iatrogenic injury. In a patient with a duplicated vas deferens, this anatomic variant may not be recognized during surgery, resulting in intraoperative injury and subsequent complications.[2]

In cystic fibrosis, abnormalities of the vas deferens, ranging from obstruction to complete absence, are invariably present.[10] Agenesis of the vas deferens is found to be associated with renal dysgenesis in patients who do not have cystic fibrosis.[12] The evaluation of the urinary system with abdominal ultrasound and sweat chloride test for cystic fibrosis were found to be normal in our patient.

A review of the English language literature revealed a total of 10 cases of vas deferens duplications including our presented case [Table 1]. All of the cases were diagnosed incidentally. Of these cases, three were diagnosed during inguinal hernia repair,[2,6,9] two at the vasectomy procedures,[4,5] two at varicocelectomy procedures,[1,8] one at the surgery for adenocarcinoma of the prostate,[7] one during undescended testis surgery (the present case), and one case was diagnosed during investigation for ectopic ureter.[3] The most common associated anomaly was inguinal hernia.[2,6,9]

**Table 1:** Duplications of the vas deferens

	Patient’s age	Side	Type	Associated anomaly
Gravgaard *et al*.[3]	26-year	Left	Complete	Left renal agenesis
Binderow *et al*.[2]	20-month	Left	Complete	Inguinal hernia
Carr[4]	36-year	Bilateral	Complete	None
Khoudary and Morgentaler[5]	42-year	Left	Partial	None
Damle *et al*.[6]	21-year	Left	Complete	Inguinal hernia
Akay *et al*.[1]	22-year	Left	Partial	Varicocele
Shariat *et al*.[7]	58-year	Right	Complete	None
Erdemir *et al*.[8]	19-year	Left	Complete	Varicocele
Chintamani *et al*.[9]	31-year	Right	Complete	Inguinal hernia
Present case	1-year	Left	Partial	Undescended testes

During undescended testis surgery, the possibility of a duplicated vas deferens at the level of the inguinal canal should be kept in mind to avoid inadvertent injury to this vital structure. To the best of our knowledge, this is the first report of partial duplication of the vas deferens associated with undescended testis.
